# Three Rotavirus Outbreaks in the Postvaccine Era — California, 2017

**DOI:** 10.15585/mmwr.mm6716a3

**Published:** 2018-04-27

**Authors:** Rachel M. Burke, Jacqueline E. Tate, Nora Barin, Carly Bock, Michael D. Bowen, David Chang, Rashi Gautam, George Han, John Holguin, Thalia Huynh, Chao-Yang Pan, Rebecca Quenelle, Catherine Sallenave, Cindy Torres, Debra Wadford, Umesh Parashar

**Affiliations:** ^1^Division of Viral Diseases, National Center for Immunization and Respiratory Diseases, CDC; ^2^Epidemic Intelligence Service, CDC; ^3^Long Beach City Health Department, Long Beach, California; ^4^San Mateo County Public Health, Policy and Planning, San Mateo, California; ^5^Santa Clara County Public Health Department, San Jose, California; ^6^California Department of Public Health Viral and Rickettsial Disease Laboratory, Richmond, California.

Before the introduction of rotavirus vaccine in 2006, rotavirus was the most common cause of severe diarrhea among U.S. children (*1*). Currently, two rotavirus vaccines are licensed for use in the United States, both of which have demonstrated good field effectiveness (78%–89%) against moderate to severe rotavirus illness (*2*), and the use of these vaccines has substantially reduced the prevalence of rotavirus in the United States (*3*). However, the most recent national vaccine coverage estimates indicate lower full rotavirus vaccine–series completion (73%) compared with receipt of at least 3 doses of vaccines containing diphtheria, tetanus, and pertussis antigens (95%), given on a similar schedule to rotavirus vaccines (*4*). In the postvaccine era in the United States, rotavirus activity persists in a biennial pattern (*3*). This report describes three rotavirus outbreaks that occurred in California in 2017. One death was reported; however, the majority of cases were associated with mild to moderate illness, and illness occurred across the age spectrum as well as among vaccinated children. Rotavirus vaccines are designed to mimic the protective effects of natural infection and are most effective against severe rotavirus illness (*2*). Even in populations with high vaccination coverage, some rotavirus infections and mild to moderate illnesses will occur. Rotavirus vaccination should continue to be emphasized as the best means of reducing disease prevalence in the United States.

## Outbreak 1: Child Care Center in Long Beach

In late March 2017, the Long Beach Department of Health and Human Services (LBDHHS) was notified of an outbreak of acute gastroenteritis (AGE) at a child care center. The facility provided daycare to 80 children aged 2–5 years and afterschool care to 135 additional children; 27 staff members were employed. LBDHHS emphasized hand hygiene, provided facility-cleaning recommendations consistent with those for norovirus outbreaks, and advised parents to keep ill children home for at least 48 hours after symptom resolution. At a site visit, LBDHHS provided detailed recommendations and education to staff members, and the facility later closed to perform more thorough cleaning. By April 17, 2017, a total of 27 cases of AGE among children and four cases among staff members had been reported; the classrooms for children aged 2 years and 3 years experienced the highest attack rates (43% and 37%, respectively). Five secondary cases among household contacts were reported. Symptom onset dates ranged from March 22 through April 12, 2017. Among 31 patients for whom symptom information was available, 22 (71%) had diarrhea, 17 (55%) had vomiting, 13 (42%) reported abdominal cramps, 12 (38%) had fever, and four (13%) reported nausea. Patient age ranged from 2 to 86 years (median age = 4 years). Three patients visited their primary care provider; no hospitalizations or deaths occurred. Norovirus was initially suspected to be the causative agent, but four stool specimens tested at the Long Beach Public Health Laboratory were norovirus-negative. Specimens were then sent to the California Department of Public Health (CDPH) Viral and Rickettsial Disease Laboratory (VRDL), a CaliciNet Outbreak Support Center, where all specimens tested positive by reverse-transcription polymerase chain reaction (RT-PCR) for rotavirus. These samples were genotyped as G12P[8] by CDC’s Rotavirus Surveillance Laboratory. The California immunization registry indicated that six (22%) of the 27 children with rotavirus were vaccinated, including four who were fully vaccinated. However, actual coverage might have been higher in this population because provider use of the registry is not mandated and the facility did not require proof of rotavirus vaccination for enrollment.

## Outbreak 2: Adult Assisted Living and Memory Care Facility in San Mateo

In early April 2017, the San Mateo County Division of Public Health, Policy, and Planning was notified of an outbreak of AGE at an assisted living and memory care facility housing 44 residents and employing 40 staff members. San Mateo health officials recommended standard control measures for gastrointestinal illness outbreaks (e.g., isolation and cohorting, contact precautions, suspension of group activities, promotion of handwashing, and disinfection with bleach solution or a disinfectant approved by the Environmental Protection Agency [EPA] as effective against norovirus). By April 10, 2017, nine cases had been reported, including four among residents and five among staff members. Symptom onset dates occurred during March 31–April 6, 2017. All nine patients had diarrhea, two reported abdominal cramps, and one had vomiting. Patient age ranged from 22 to 90 years (median age = 47 years); no patients were eligible to have received rotavirus vaccine. At least one patient sought primary care; no hospitalizations or deaths were reported. As in the first outbreak, norovirus was initially suspected, but two stool specimens tested at the county public health laboratory were norovirus-negative. These specimens were then sent to CDPH VRDL for additional testing, where they were both found to be rotavirus-positive by RT-PCR; they were later genotyped as G12P[8] by CDC.

## Outbreak 3: Subacute Care Facility for Children in Santa Clara County

On May 1, 2017, the Santa Clara County Public Health Department (SCCPHD) was notified of an outbreak of AGE at a subacute inpatient care facility for patients aged <21 years with complex medical needs. In consultation with SCCPHD, the facility increased cleaning and disinfection with bleach solution, implemented cohorting and isolation procedures, cancelled group activities, and suspended new admissions. A site visit by SCCPHD confirmed good adherence to hand hygiene and contact precautions. Nonetheless, by the end of the outbreak, 24 of the 25 facility patients and three of 115 staff members had fallen ill. Symptom onset dates ranged from April 24 through May 17, 2017. The median duration of symptoms was 7.5 days; 23 (85%) patients had diarrhea, and 15 (56%) had vomiting. Patient age ranged from 6 months to 39 years (median age = 2 years). Although most cases resolved without major complications, one child aged 22 months with preexisting respiratory failure died; the cause of death was attributed to rotavirus-induced dehydration. This patient, as well as 16 others, had received no doses of rotavirus vaccine; three other patients had received a single dose. Though reasons for nonvaccination were not tracked by the facility, many of the children had been vaccinated according to delayed vaccination schedules and might have aged out of eligibility for rotavirus vaccination (*1*).* Laboratory testing by a gastroenteritis multipathogen PCR panel at a local hospital confirmed rotavirus in 11 of 14 samples; no other pathogens were detected. Eight samples forwarded to CDPH VRDL were found to be PCR-positive for rotavirus, and five were then forwarded to CDC for genotyping. Four were genotyped as G12P[8]; one was identified as a G12 virus, but its P type was not identified.

## Discussion

Although U.S. rotavirus activity has substantially declined since the introduction of rotavirus vaccine, rotavirus disease continues to occur sporadically throughout the year and epidemically in a biennial winter-spring seasonal pattern, affecting even vaccinated persons (*3*). Further, as evidenced by these outbreaks as well as previously published reports, rotavirus affects not only young children, but also adults, especially those in congregate living settings (*5*,*6*).

Although these outbreaks represent a small proportion of U.S. rotavirus outbreaks, and are not necessarily representative of all outbreaks, they illustrate some general characteristics of rotavirus outbreaks in the postvaccine era. The first outbreak, in the child care center, was characterized by comparatively mild illness in otherwise healthy children and illustrates that rotavirus outbreaks do occur in the postvaccine era, even among healthy, vaccinated populations. Current rotavirus vaccines are highly effective against severe diarrheal illness, but they do not necessarily prevent infection or milder disease. Thus, rotavirus disease and outbreaks can occur even in populations where vaccination coverage is high.

The second outbreak, in an adult assisted living and memory care facility, demonstrated that rotavirus can and does cause illness in adult populations and can spread easily among adults living in close quarters, such as nursing homes. Though adults do not receive rotavirus vaccine, research has indicated that rotavirus vaccination of children might have an indirect protective effect in the adult population (*7*). As use of multipathogen PCR testing increases, there might be more detection of rotavirus outbreaks in adult populations. Rotavirus outbreaks are sometimes initially suspected to be norovirus, but rotavirus should not be ruled out as a causative agent because of the age of the affected population. Nonetheless, the public health recommendations are similar for both norovirus and rotavirus. Hand hygiene, cohorting and isolation, and surface disinfection with appropriate products should be emphasized. Cleaning surfaces with soap and water followed by a 5-minute application of 1000–5000 ppm chlorine solution (5–25 tbsp [2.5–12.5 oz] of household bleach [5.25% sodium hypochlorite] per gallon of water) or other disinfectant registered as effective against norovirus by the EPA is appropriate for both pathogens (*8*,*9*).

The third outbreak also occurred among children; however, in contrast to the first outbreak, it affected an already vulnerable population with low vaccination coverage and was associated with the highest attack rate among the three outbreaks, as well as one fatality. Reasons for nonvaccination among children in the facility were not ascertained; however, most patients at the facility had spent time in neonatal and pediatric intensive care units, where use of live viral vaccines is discouraged (*1*), and many were too old to begin rotavirus vaccine after discharge. Additional research might be necessary to evaluate the risks and benefits of this practice.

Although the impact of rotavirus vaccines against various commonly circulating strains has been well documented, rotavirus outbreaks will continue to occur, even among highly vaccinated populations. Although genotype G12P[8] was detected in samples from all three outbreaks, this likely reflects the fact that G12P[8] has been the most common circulating strain in the United States in recent years (*10*). Both currently available rotavirus vaccines have demonstrated effectiveness against this strain (*2*); however, because rotavirus vaccination coverage lags behind that of other childhood vaccines, many children remain susceptible to severe rotavirus disease. Public health practitioners, as well as clinicians, should continue to consider rotavirus as a suspected agent in cases of AGE across all ages and should promote rotavirus vaccination among eligible infants according to CDC recommendations.

**Summary**

**What is already known about this topic?**

The introduction of vaccines against rotavirus, the most common cause of severe diarrhea among U.S. children, has substantially reduced disease incidence.

**What is added by this report?**

Rotavirus outbreaks in a child care center and an adult assisted living facility caused primarily mild illness. In a pediatric subacute care facility, illness was widespread and resulted in one death in a toddler with underlying complications.

**What are the implications for public health practice?**

Rotavirus vaccination is most effective against severe disease. Vaccination reduces transmission and might confer indirect protection to unvaccinated individuals, but outbreaks will continue. Public health practitioners and clinicians should consider rotavirus in cases of acute gastroenteritis and promote rotavirus vaccination per CDC guidelines.

## Footnotes

* The maximum ages for initiating and completing the rotavirus vaccination series are 14 weeks 6 days and 8 months, respectively.
